# Transmission potential of *Mayaro virus* by *Aedes albopictus*, and *Anopheles quadrimaculatus* from the USA

**DOI:** 10.1186/s13071-020-04478-4

**Published:** 2020-12-09

**Authors:** Constentin Dieme, Alexander T. Ciota, Laura D. Kramer

**Affiliations:** 1 Wadsworth Center, New York State Department of Health, Slingerlands, New York USA; 2 Department of Biomedical Sciences, School of Public Health, State University of New York at Albany, Albany, New York USA

**Keywords:** *Mayaro virus*, *Aedes albopictus*, *Anopheles quadrimaculatus*, Vector competence

## Abstract

**Background:**

*Mayaro virus* (MAYV; *Alphavirus, Togaviridae*) is an emerging pathogen endemic in South American countries. The increase in intercontinental travel and tourism-based forest excursions has resulted in an increase in MAYV spread, with imported cases observed in Europe and North America. Intriguingly, no local transmission of MAYV has been reported outside South America, despite the presence of potential vectors.

**Methods:**

We assessed the vector competence of *Aedes albopictus* from New York and *Anopheles quadrimaculatus* for MAYV.

**Results:**

The results show that *Ae. albopictus* from New York and *An. quadrimaculatus* are competent vectors for MAYV. However, *Ae. albopictus* was more susceptible to infection. Transmission rates increased with time for both species, with rates of 37.16 and 64.44% for *Ae. albopictus*, and of 25.15 and 48.44% for *An. quadrimaculatus*, respectively, at 7 and 14 days post-infection.

**Conclusions:**

Our results suggest there is a risk of further MAYV spread throughout the Americas and autochthonous transmission in the USA. Preventive measures, such as mosquito surveillance of MAYV, will be essential for early detection.

## Background

*Mayaro virus* (MAYV; *Togaviridae, Alphavirus*) is an emerging virus first isolated in Trinidad in 1954 from the serum of febrile patients. *Mayaro virus* strains are grouped into three distinct genotypes: L (limited), N (new), and D (widely dispersed) [1–4]. Similar to other medically important alphaviruses, MAYV is a mosquito-borne arbovirus that causes fever, headache, myalgia, rash, and arthralgia of large joints and, occasionally, arthritis in humans [5]. New World primates of the families Cebidae and Callithricidae are considered to be potential natural reservoirs for the virus [6, 7]. The virus has also been found in a migrating bird, equids, anteaters, armadillos, opossums, and rodents [8, 9].

Endemic in South America countries, the frequency of Mayaro virus disease in humans has increased in number in recent years, and imported cases have been detected in previously unaffected areas, such as Europe and the USA [3]. Further expansion of MAYV range could be facilitated by global climate change, rapid urbanization and higher mobility of the population, lack of effective vector control, and spreading of vector populations to new geographic regions [5, 10, 11]. Different mosquito species have been found to be infected with the virus, including *Mansonia venezuelensis*, *Haemagogus janthinomys*, *Sabethes* spp., and *Culex* spp. [7, 10]. Moreover, *Aedes albopictus*,* Aedes aegypti*,* Anopheles gambiae*,* Anopheles stephensi*,* Anopheles quadrimaculatus*, and *Culex quinquefasciatus* are known to be competent vectors of MAYV [12–14].

Many travelers from MAYV endemic areas visit New York each year; however, to date there is no information available on the potential of local mosquitoes to transmit MAYV. To evaluate this risk, we infected *Ae. albopictus* (temperate strain) and *An. quadrimaculatus* with MAYV and evaluated their capacity to transmit the virus. Our results show that both mosquito species are competent vectors of MAYV, with *Ae. albopictus* being the more efficient vector.

## Methods

### Mosquitoes

A colony of unknown generations of *An. quadrimaculatus* (Orlando strain) was obtained from BEI Resources (MRA-139; https://www.beiresources.org/) and maintained at 27 °C under standard rearing conditions [15]. Larvae were maintained in plastic rectangular flat containers [35.6 × 27.9 width × 8.3 cm (length × width × height); Sterilite Corp., Townsend, MA, USA, catalogue no. 1963] at a density of 150–200 larvae per liter of water and fed with Tetra pond Koi growth food. Food was renewed every 2–3 days until adult emergence. After emergence, adults were kept in 8 × 8 × 8 in. (20.3 cm) metal cages (Bioquip Products Inc., Compton, CA, USA) under controlled conditions (27 ± 1 °C; 70% relative humidty; 12:12-h light:dark photoperiod) and fed with 10% sucrose solution ad libitum until their use in experiments. The *Ae. albopictus* colony (Spring Valley, NY, USA; kindly provided by Laura Harrington, Cornell University) was newly established in 2019 from field-collected eggs. *Aedes albopictus* were hatched in distilled water, reared, and maintained similarly to the *Anopheles* described above. F4 females were used for the MAYV challenge experiments.

### Mosquito vector competence for *Mayaro virus*

*Mayaro virus* strain TRVL-4675 (isolated from the serum of an infected human in Trinidad in 1954 and belonging to the D genotype) was freshly propagated in Vero (African Green Monkey kidney) cell cultures maintained at 37 °C, 5% CO_2_. At 48 h following infection (multiplicity of infection ≈ 1.0), the supernatant was harvested and diluted 1:1 with defibrinated sheep blood plus a final concentration of 2.5% sucrose. For each species, three biological replicates at different times were performed, and for each experiment 90–100 female mosquitoes were allowed to feed. Female *An. quadrimaculatus* mosquitoes (3–5 days old) deprived of sugar for 1–2 h and female *Ae. albopictus* (5–7 days old) deprived of sugar for 24 h were allowed to feed on MAYV–blood suspension for 45 min via a Hemotek membrane feeding system (Discovery Workshops, Acrington, UK) with a porcine sausage casing membrane, at 37 °C [15]. Following feeding, females were anesthetized with CO_2_, and fully engorged mosquitoes were transferred to 0.6-L cardboard containers and maintained with 10% sucrose at 27 °C until harvested for testing. Aliquots (1 mL) of each blood meal pre-feeding were frozen at − 80 °C to determine MAYV titer by plaque assay on Vero cells.

### Detection of *Mayaro virus*

Infection, dissemination, and transmission were determined on days 7 and 14 post-infectious blood meal (dpi: days post-infection), as previously described [15]. Blood meals, mosquito bodies, legs, and salivary secretions were assayed for infection by plaque assay on Vero cells [16]. Briefly, Vero cells were seeded in six-well plates at a density of 6.0 × 10^5^ cells per well and incubated for 3–4 days at 37 °C, 5% CO_2,_ to produce a confluent monolayer. The cell monolayers were inoculated with 0.1 mL of 10-fold serial dilutions of the blood meals (diluted in BA-1) in duplicate or with undiluted mosquito bodies, legs, and salivary secretions from each homogenized mosquito sample. Viral adsorption was allowed to proceed for 1 h at 37 °C with rocking of the plates every 15 min. A 3-mL overlay of MEM, 5% fetal bovine serum, and 0.6% Oxoid agar supplemented with 0.2× penicillin–streptomycin/mL, 0.5 μg of fungizone (amphotericin B)/mL, and 20 μg of gentamicin/mL was added at the conclusion of adsorption. The infected monolayers were incubated at 37 °C, 5% CO_2_. After 2 days of infection a second overlay, similar to the first but with the addition of 1.5% Neutral Red (Sigma–Aldrich Co., St. Louis, MO), was added to the wells, and the plates were incubated overnight at 37 °C, 5% CO_2_. For the blood meal, the plaques were counted, and the viral titer was calculated and expressed as plaque-forming units per milliliter. For mosquito samples, presence or absence of plaques was checked.

Dissemination rate was defined the proportion of mosquitoes with infected legs among the mosquitoes with infected bodies and transmission rate as the proportion of mosquitoes with infectious saliva collected by capillary transmission method [15] among mosquitoes with disseminated infection. Dissemination efficiencies and transmission efficiencies refer to the proportion of mosquitoes with infectious virus in the legs or in the saliva, respectively, among all mosquitoes that fed.

### Statistical analysis

A Fisher’s exact test was used to compare combined infection rates, dissemination rates, dissemination efficiencies, transmission rates, and transmission efficiencies between or within mosquito species and between time points. All statistical analyses were carried out at a significance level of *P* < 0.05. OpenEpi, version 3, an open source calculator (TwobyTwo; https://www.openepi.com/TwobyTwo/TwobyTwo.htm), was used for all statistical analysis.

## Results

A total of 180 *An*. *quadrimaculatus* and 180 *Ae. albopictus* were analyzed in this study.

Oral challenge with MAYV led to the establishment of high infection rates in both mosquito species. The mean infection rates of *Ae. albopictus* and *An. quadrimaculatus* were significantly different at both time points [7 dpi: 100.0 vs. 82.22%, respectively, *P* < 0.0001, odds ratio (OR) 38.7, 95% confidence interval (CI) 2.281–656.8; 14 dpi: 100.0 vs 74.44%, respectively, *P* < 0.0001, OR 61.45, 95% CI 3.665–1030; Table 1]; however, no significant difference between time points within mosquito species was observed.

**Table 1:** *Aedes albopictus* and *Anopheles quadrimaculatus* infection rates, dissemination rates, and transmission rates after exposure to *Mayaro virus*

Mosquito species	Replicate	Blood meal titer (log_10_ PFU/ml)	7 dpi						14 dpi					
			*N* tested	Infection rate,* N *(%)	Dissemination rate,* N* (%)	Dissemination efficiency,* N* (%)	Transmission rate, N (%)	Transmission efficiency,* N* (%)	*N* tested	Infection rate,* N* (%)	Dissemination rate,* N* (%)	Dissemination efficiency, N (%)	Transmission rate, N (%)	Transmission efficiency,* N* (%)
*Ae. albopictus*	1	8.23	30	30 (100.0)	29 (96.67)	29 (96.67)	13 (44.82)	13 (43.33)	30	30 (100.0)	30 (100.0)	30 (100.0)	24 (80.0)	24 (80.0)
	2	8.14	30	30 (100.0)	30 (100.0)	30 (100.0)	10 (33.33)	10 (33.33)	30	30 (100.0)	30 (100.0)	30 (100.0)	21 (70.0)	21 (70.0)
	3	7.97	30	30 (100.0)	27 (90.0)	27 (90.0)	9 (33.33)	9 (30.00)	30	30 (100.0)	30 (100.0)	30 (100.0)	13 (43.33)	13 (43.33)
	Mean ± SD	NA	30	100.0	95.56 ± 5.09	95.56 ± 5.09	37.16 ± 6.63	35.55 ± 6.94	30	100.0	100.0	100.0	64.44 ± 18.96	64.44 ± 18.96
*An. quadrimaculatus*	1	8.06	30	25 (83.33)	19 (76.0)	19 (63.33)	8 (42.11)	8 (26.66)	30	21 (70.00)	16 (76.19)	16 (53.33)	7 (43.75)	7 (23.33)
	2	7.87	30	25 (83.33)	8 (32.0)	8 (26.67)	0 (0.0)	0 (0.0)	30	19 (63.33)	7 (36.84)	7 (23.33)	4 (57.14)	4 (13.33)
	3	8.39	30	24 (80.0)	18 (75.0)	18 (60.0)	6 (33.33)	6 (20.0)	30	27 (90.00)	18 (66.67)	18 (60.00)	8 (44.44)	8 (26.67)
	Mean ± SD	NA	30	82.22 ± 1.92	61.0 ± 2 5.12	50.0 ± 20.27	25.15 ± 22.22	15.55 ± 13.88	30	74.44 ± 13.88	59.9 ± 20.53	45.55 ± 19.53	48.44 ± 7.54	21.11 ± 6.94

We used the infection rates, dissemination rates, and transmission rates for comparison within mosquito species. A significant difference within mosquito species was only observed for *Ae. albopictus* transmission [*P* < 0.0001, odds ratio (OR) 0.3269, 95% conficence interval (CI) 0.1769–0.6043]. Significant differences were observed between *Ae. albopictus* and *An. quadrimaculatus* infection rates (*P* < 0.0001, OR 38.7, 95% CI 2.281–656.8 and *P* < 0.0001, OR 61.45, 95% CI 3.665–1030 , at 7 dpi and 14 dpi, respectively) and dissemination efficiencies at both time points (*P* < 0.0001, OR 21.5, 95% CI 7.27–63.58 and *P* < 0.0001, OR 213.9, 95% CI 12.88–3554, at 7 dpi and 14 dpi, respectively). Between species, the transmission efficiencies were significantly different at both time points (*P* < 0.0017, OR 2.995, 95% CI 1.465–6.122 at 7 dpi and *P* < 0.0001, OR 6.773, 95% CI 3.482–13.17 at 14 dpi)

dpi, Days post-infection; NA, not applicable, PFU, plaque-forming units; SD standard deviation

Within mosquito species similar mean dissemination rates were observed for *Ae*. *albopictus* and *An. quadrimaculatus* for both time points (95.56% at 7 dpi vs. 100% at 14 dpi and 61.0% at 7 dpi vs. 59.9% at 14 dpi, respectively; Table 1). Dissemination efficiencies at 7 and 14 dpi were significantly different between mosquito species (*P* < 0.0001, OR 21.5, 95% CI 7.27–63.58 and *P* < 0.0001, OR 213.9, 95% CI 12.88–3554, at 7 dpi and 14 dpi, respectively; Table 1). Detection of infectious viral particules in mosquitoes collected at 7 and 14 dpi indicated that *Ae*. *albopictus* and *An. quadrimaculatus* are highly susceptible to MAYV through oral challenge and subsequently support viral replication.

Infectious viral particules were detected in saliva of individuals with disseminated infections for 37.16 and 64.44% *Ae. albopictus* and for 25.15 and 48.44% *An. quadrimaculatus*, at 7 and 14 dpi, respectively (Table 1). In both mosquito species, the transmission rates increased with time; however, a significant difference was only observed for *Ae. albopictus* (*P* < 0.0001, OR 0.3269, 95% CI 0.1769–0.6043; Table 1). Furthermore, transmission efficiencies were significantly different between mosquito species at both time points (*P* < 0.0017, OR 2.995, 95% CI 1.465–6.122 and *P* < 0.0001, OR 6.773, 95% CI 3.482–13.17, at 7 dpi and 14 dpi, respectively; Table 1).

## Discussion

To our knowledge, this is the first study to examine the vector competence of a temperate population of *Ae*. *albopictus* from the Northeast USA, and the second study on *An. quadrimaculatus*, a native and abundant anopheline mosquito in the Northeast USA, including New York, for MAYV. As mosquitoes and their viruses continue to expand their geographic range and emerge in unpredictable ways, the USA could face an increased threat from MAYV in the future. Our data demonstrate that New York *Ae. albopictus* and *An. quadrimaculatus* are highly competent vectors of MAYV.

When multiple mosquito species are involved in the transmission of an arbovirus, the effort needed to prevent human exposure increases. Determining the role of each species is important [17]. We found that dissemination and transmission rates were lower for *An. quadrimaculatus* than for *Ae. albopictus*. In locations where *Ae. albopictus* is prevalent, this difference might play a role in the epidemiology of MAYV considering its high vector competence, were it to be introduced.

*Aedes albopictus* is a highly invasive species that has been introduced into the USA where it has become permanently established in at least 27 states, including New York [18, 19]. It is predicted that this mosquito species will continue to spread globally over the coming decades, increasing the risk to human health [20]. In the USA, *Ae. albopictus* may be infected with a number of arboviruses, including *Eastern Equine Encephalitis virus*, *Dengue virus*, *St. Louis encephalitis virus*, *La Crosse orthobunyavirus*, and *West Nile virus* [19]. In addition, its role as a vector is recognized for *Chikungunya virus* and *Zika virus*, both introduced recently into the USA [19, 20]. Using a temperate population of *Ae. albopictus* from New York, we confirmed earlier studies that demonstrated the potential of *Ae. albopictus* to transmit MAYV [13, 14, 21].

The high infection rates (85–100%) obtained in our results are similar to the reports of others [13, 14]. Moreover, the high dissemination and transmission rates observed in our study corroborate the findings of Diop et al [13]. However, Wiggins et al [14], using the same MAYV strain that we used, found lower transmission rates compared to our study and Pereira et al. [21]. These differences could be due to the genetic background (geographical origin) of the vector and/or the difference in mosquito incubation temperature, as has been shown for *Chikungunya virus* [22, 23].

*Anopheles* mosquitoes are persistently exposed in nature to diverse arboviruses, but in general an assessment of their potential to transmit arboviral pathogens has been neglected. In addition to MAYV, vector competence of *Anopheles* mosquitoes for *O’nyong nyong* (ONNV) *virus*, *Rift Valley fever phlebovirus*, *Eastern equine encephalitis virus*, and *Cache Valley orthobunyavirus* has been reported [12, 24–26]. However, only ONNV is known to rely on *Anopheles* spp. as primary vectors [27, 28]. *Anopheles quadrimaculatus* are primarily mammalophagic mosquitoes. In the Northeast USA, white-tailed deer are the predominately identified vertebrate host [29]. However, this may be an artefact of human accessibility rather than an indication of preference. *Anopheles quadrimaculatus* mosquitoes are historically important vectors of human malaria parasites (*Plasmodium vivax*) [30], suggesting that they have a high level of anthropophily. Furthermore, white-tailed deer overabundance and availability throughout the region may explain mosquitoes feeding behavior [17, 31]. It is suggested that *An. quadrimaculatus* and *An. punctipennis* may contribute to the transmission of Eastern equine encephalitis, Jamestown Canyon, and Cache Valley viruses in the Northeast USA [29]. Recently, the capacity of *An*. *quadrimaculatus* to transmit MAYV at 7 dpi but not at 14 dpi was demonstrated [12]. In our study, *An*. *quadrimaculatus* mosquitoes were able to transmit the virus at both time points, suggesting this species may be an overlooked vector for MAYV emergence and invasion in the USA.

## Conclusion

Information on the competence of mosquito vectors is essential for controlling and preventing viruses transmitted by arthropods. While it is not possible to accurately predict the emergence of a disease, in light of our results, MAYV presents a health threat to the USA, and local authorities should reinforce epidemiological and entomological surveillance to detect the introduction of this viral pathogen.

## Data Availability

Data generated in this study are available from the corresponding authors upon reasonable request.

## Abbreviations

dpi: Days post-infection
MAYV: *Mayaro virus*
ONNV: *O’nyong nyong virus*
